# Prevalence of cystic echinococcosis in relatives of patients undergoing surgery for hepatic cystic echinococcosis in an endemic region

**DOI:** 10.1371/journal.pntd.0011813

**Published:** 2023-12-08

**Authors:** Carlos Manterola, Josue Rivadeneira, Claudio Rojas, Tamara Otzen

**Affiliations:** 1 Center for Morphological and Surgical Studies. Universidad de La Frontera. Chile; 2 PhD. Program in Medical Science, Universidad de La Frontera, Chile; 3 Núcleo Milenio de Sociomedicina. Santiago, Chile; 4 Zero Biomedical Research, Quito, Ecuador; Istituto Superiore Di Sanita, ITALY

## Abstract

**Background:**

Cystic echinococcosis (CE) is an endemic disease in southern Chile. The aim of this study was to ascertain the prevalence of CE among relatives of patients who underwent surgical intervention for this disease in Cautín, a province of southern Chile.

**Methodology/principal findings:**

Cross-sectional study. Relatives of patients who underwent surgery for hepatic echinococcosis (HE), who lived at the same address, during the period 2000–2020 were studied. A total of 288 relatives of 322 patients who underwent surgery for HE participated in a CE screening. All these relatives were interviewed and underwent abdominal ultrasonography, chest X-ray and immunodiagnostic studies (relatives who had been diagnosed with or had undergone surgery for CE were excluded). Descriptive statistics were applied. Prevalence calculation, odds ratio (OR), and their respective 95% confidence intervals (95% CI) were determined. Abdominal or thoracic CE was verified in 42 relatives of subjects operated on for HE (mean age 41±8 years; 73.8% women; 38.1% of cases had two or more cysts), all of them new and asymptomatic cases. CE was detected in the lungs, liver, peritoneum, and spleen in 16.7%; 71.4%; 7.1%; and 4.8%, respectively. The overall prevalence of EQ during the studied time period was 14,6% (17.9% and 12.3% in relatives of first and second degree respectively (OR:1.56; CI 95%: 0.81; 3.01).

**Conclusion/significance:**

There is a high prevalence of CE in relatives of patients undergoing surgery by this disease in the province of Cautín, Chile.

## Author summary

Cystic echinococcosis (CE), caused by the cestode *Echinococcus granulosus*, is a neglected tropical zoonotic disease that predominantly affects impoverished pastoral communities worldwide. It is endemic in the southernmost regions of South America, specifically in Argentina, Chile, Peru, and Uruguay, with over 5000 new human cases reported annually across the four countries. The dog-sheep cycle has been identified as the most epidemiologically significant, as the parasite circulates between farm dogs and livestock, associating with cattle breeding and the feeding of dogs with infected offal. In the 21st century, remarkable progress has been achieved in the fields of genetics, genomics, and molecular epidemiology of causative parasites, as well as in diagnostic techniques, treatment modalities, and control strategies, including the development and distribution of vaccines. Nevertheless, there remain several unresolved issues, one of which is the prevalence among relatives of patients undergoing surgery for CE, because there is limited evidence regarding the infection of relatives of operated patients.

## Introduction

Cystic echinococcosis (CE) constitutes an endemic zoonotic disease in Chile, affecting both humans and ungulates due to infection with the larval stage of the tapeworm *Echinococcus granulosus sensu lato*. The disease’s incidence was recorded at a rate of 1.9 cases per 100,000 inhabitants in 2019 [1]. Within the regions of La Araucanía and Los Ríos, even higher rates are reported: 6.1 cases per 100,000 inhabitants, with mortality rates of 0.1 per 100,000 inhabitants and a case fatality rate of 1.3% [2–4], as evidenced in prior studies. Over the course of time, the prevalence of CE in the La Araucanía region has persistently exceeded national averages, reaching rates up to 30 cases per 100,000 inhabitants in recent years [5]. The annual costs associated solely with surgical interventions are estimated at approximately USD 2.46 million. Upon inclusion of expenditures related to medical leaves and the consequent losses in productivity, the cumulative economic impact on human health approximates USD 3.13 million [3].

The initial reports of CE in relatives of patients afflicted by this disease were presented by Thorstensen and Krabbe in 1840 and 1866, respectively [6]. Subsequently, a case series involving family members affected by CE with hepatic and pulmonary involvement was published [7]. Later, a study encompassing 29 families with CE was documented, revealing that 59% of cases were identified among siblings [8]. Since then, instances of CE affecting both nuclear and extended families have been documented. It is noteworthy that the transmission of the definitive host to humans occurs via the fecal-oral route and no reports have ever found genetic traits that predispose to the infection [9]. Nonetheless, transmission within family groups is seldom addressed in the literature, with scarce reports such as those by Musio, who documented EQ infection in seven out of 11 members of an Athenian family in Greece [9]; Karadağlı, reporting CE in four family members [10]; Yang, who observed a prevalence of 9% for CE and 5.9% for alveolar echinococcosis (a combined prevalence of 14.9%) among individuals from Nanwan, Ningxia Hui, China. This study involved an Islamic community comprising 167 members across four families, wherein 12 family members died to CE [11]; and Larrieu in a case-control study of the risk factors for CE among children in Rio Negro, Argentina, founded that the main risk factor significantly associated with CE were having a family member with the disease (OR = 3.11; CI = 0.92–10.47) [12].

The aim of this study was to ascertain the prevalence of CE among relatives of patients who underwent surgical intervention for this disease in Cautín, a province of southern Chile.

## Methods

### Ethics statement

The study adhered to ethical research guidelines set forth by the Helsinki Declaration [13]. Anonymization procedures were employed to mask the identities of patients and their family members. Verbal informed consent was obtained from all family members involved in the study. The study was approved by Institutional Review Board Scientific Ethics Committee Clínic Mayor 15/2021.

The report of the results of this study was made following the MInCir-DOS Declaration for reporting Descriptive Observational Studies [14].

### Study design

Cross-sectional study.

### Setting

The study was conducted at the Regional Hospital and RedSalud Mayor Clinic in Temuco, Chile. The recruitment period spanned from January 2000 to December 2020.

### Participants

All first- or second-degree relatives who shared a household with the index case, surgically treated for liver CE by the primary author, were included. Participants were recruited from the aforementioned centers and within the specified time frame. Moreover, these participants originated from the Cautín province. Those relatives who had been diagnosed with or had undergone surgery for CE were excluded from the study.

### Sample size

The determination of sample size was not undertaken.

### Screening

In the year 2022, study participants underwent a comprehensive assessment including abdominal ultrasonography, chest radiography, and immunodiagnostic evaluations (ELISA-IgG and ELISA-IgE, ISP-Chile). These diagnostic procedures were meticulously recorded in a dedicated registration document.

### Variables

The primary outcome variable centered on the imaging diagnosis of either thoracic or abdominal CE. Furthermore, an array of additional variables of significance were considered, encompassing demographic factors such as gender and age at the time of diagnosis, along with clinical characteristics such as number of cysts, specific location of the CE (by organs), size of cyst(s), and the outcomes from immunodiagnostic analyses (ELISA-IgG and ELISA-IgE).

### Statistical analysis

Data collection and analysis were conducted using Stata 12.0 software. An initial exploratory data analysis was performed. Shapiro-Wilk test, for sample size less than 50 observations was used to assess data distribution. Descriptive statistics were employed, encompassing measures of central tendency and dispersion (including means, standard deviations, minimum and maximum values). Additionally, prevalence rate, odds ratios (OR), and their corresponding 95% confidence intervals (95% CI) were computed.

### Bias

To minimize potential information biases, a blinded data collection approach was implemented for both patients and their respective family members.

## Results

**Fig 1:**
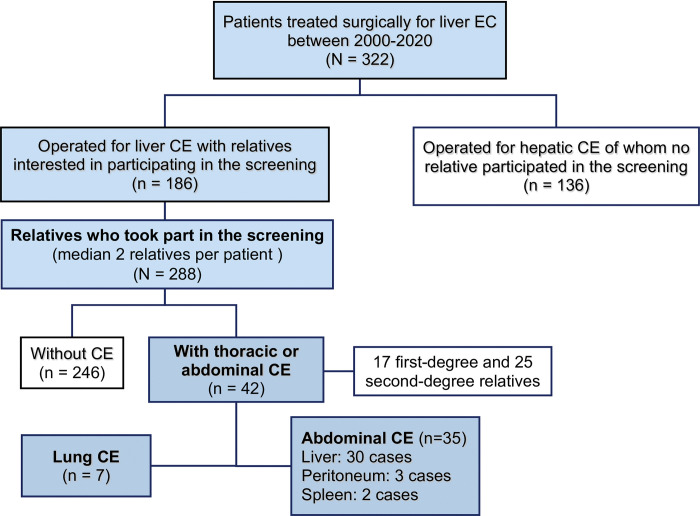
Flowchart of the participants.

During the study period, a total of 322 patients underwent surgical treatment for liver CE (no cases of thoracic CE neither CE treated by medical therapy or with inactive cysts was considered). Among these patients, 288 relatives opted to participate in the screening program. Out of these, 186 patients contributed with a family member, 55 had relatives who chose to participate (median two relatives per patient), in 246 of which the existence of CE was not verified (Fig 1). The presence of thoracic or abdominal CE was confirmed in 42 of the screened relatives (17 first-degree and 25 second-degree relatives), yielding a prevalence of 14.6%.

Regarding the anatomical distribution of confirmed CE cases, the liver was the most prevalent site (30 cases; 71.4%), followed by the lung (seven cases; 16.7%), peritoneum (three cases; 7.1%), and spleen (two cases; 4.8%), as illustrated in Fig 1.

The mean age of the screened individuals was 41±8 years, with 31 (73.8%) being female. Comorbidity was observed in 14 cases (33.3%), as detailed in Tables 1 and 2.

**Table 1:** Relatives of patients surgically treated for liver CE. Characteristics observed in the screening. (N = 42).

Variable	Average ± SD	Minimum	Maximum
Age (years)	41 ± 8	16	73
Hematocrit (%)	38 ± 3	30	45
Leucocytes (cell/mm^3^)	7704 ± 2600	4300	13600
Bilirubin (mg/dL)	0.8 ± 0.5	0.4	1.5
Alkaline phosphatase (U/L)	358 ± 197	120	820
Aspartate aminotransferase (U/L)	34 ± 6	9	167
Alanine aminotransferase (U/L)	40 ± 9	9	293
Cyst diameter (cm)	9.3 ± 2.5	5	13

**SD:** Standard Deviation

**Table 2:** Relatives of patients surgically treated for liver CE. Characteristics observed in the screening. (N = 42).

Variable	N° cases	%
**Gender**		
Feminine	31	73.8
Masculine	11	26.2
**Associated morbidity**		
Yes *	14	33.3
No	28	66.7
**ELISA-IgE** **		
Positive	10	23.8
Negative	32	76.2
**ELISA-IgG** **		
Positive	11	26.2
Negative	31	73.8
**Number of cysts**		
One	26	61.9
Two or more #	16	38.1
**Type of cysts (main lesion)** ##		
CE1	12	28.6
CE2	15	35.7
CE3a	15	35.7

*: Cholelithiasis (6 cases), asthma (2 cases), malnutrition (2 cases), pregnancy (2 cases), hypertension (1 case), and type 2 diabetes mellitus + hypertension (1 case).

**: No association was observed between positivity of the test and type of cyst (CE1, CE2 and CE3a).

#: Three relatives presented with 3 cysts each.

##: Peritoneal and splenic cysts were entirely of CE1 type; pulmonary cysts were classified as CE2 (4 out of 7) and CE3a (3 out of 7); hepatic cysts were classified as CE1 (7 out of 30), CE2 (11 out of 30), and CE3a (12 out of 30).

The results of the general diagnostic tests were within their respective normal ranges on average. However, abnormal liver function tests were observed in five relatives (Table 1). Immunodiagnostic tests yielded negative results in over 70% of cases (Table 2). The average cyst diameter was 9.3±2.5, with two or more cysts observed in 38.1% of cases (Tables 1 and 2). The distribution by cyst type (taking into consideration the primary lesion) is detailed in Table 2; however, in all cases, cysts of type CE1, CE2, and CE3a were observed.

Seventy-one point four percent of the primary cysts were situated in the liver, 16.7% in the lungs, 7.1% in the peritoneum, and 4.8% in the spleen. The overall prevalence of CE during the studied time period was 14.6% (17.9% among first-degree relatives and 12.3% among second-degree relatives). OR was 1.56 (95% CI: 0.81; 3.01).

All these cases underwent surgical treatment upon diagnosis confirmation. No morbidity or mortality was recorded, and with an average follow-up period of 97.1±5.9 months, a single case of recurrence (2.3%) was observed, which was operated on two months after the initial diagnosis.

## Discussion

Evidence regarding CE prevalence among relatives of patients undergoing hepatic CE surgery is limited (Table 3). Upon reviewing the available studies, it becomes evident that individuals without prior CE diagnosis, who cohabited with patients who had undergone CE surgery, exhibited a prevalence of 12.7% following serological assessments, chest radiography, and abdominal ultrasound examinations [15].

**Table 3:** CE in relatives of patients with liver CE.

Author, year	Country of origin	N° of cases with CE	N° of relatives infected	Infected organs
Musio, 1989 [9]	Greece	7	11	Lungs, liver and kidneys
Karadağlı, 2015 [10]	Turkey	4	5	Lungs, liver, spleen and kidneys
Yang, 2006[11]	China	13	221	Liver
Cobanoğlu, 2012 [14]	Turkey	13	102	Lungs, liver and kidneys
Eilbigi, 2020 [15]	Afghanistan	33	214	Lungs, liver and kidneys
Villa Micó, 2021 [16]	Argentina	5	67	Abdominal
Kia, 2021 [21]	Iran	96	7	Liver
Ramírez, 2018 [23]	Peru	15	15	Lungs, brain and liver
Capello, 2013 [24]	Italy	10	32	Lungs and liver

In a recent study involving relatives of 214 CE patients who underwent surgery in Afghanistan, hepatic, pulmonary, and splenic cysts were confirmed in eight, two, and one family member respectively. Positive CE serologies were detected in 22 relatives, yielding a prevalence of 10.2% following ELISA antibody detection, chest radiography, and abdominal ultrasound [16]. In another ultrasound-based screening study conducted in La Rioja, Argentina, it was concluded that the prevalence of relatives of surgically treated CE patients was 7.5% [17]. Therefore, the prevalence uncovered in our study experience (14.6%) is somewhat higher than that reported in previous studies.

Nonetheless, there appears to be no association between relatives with CE and the involved viscera. As illustrative instances, there are indications of families and relatives akin to those described, who have exhibited combinations of CE with pulmonary, hepatic, and other organ involvement [18,19]. However, reports also exist of infection affecting identical organs within families encompassing solely the lung [20] or the liver [21].

Furthermore, in a cross-sectional study designed to investigate the prevalence of CE among first-degree relatives of patients infected and surgically treated in Mashhad, Iran (n = 46), serological assessments, abdominal ultrasound, and chest radiography were conducted on all family members (n = 114). This investigation revealed that seven participants from five families (6.1%) were infected [22]. In another study known as the HERACLES project, a semi-structured questionnaire on CE risk factors was administered to 24,687 individuals from rural areas in Bulgaria, Romania, and Turkey. This inquiry highlighted that being a homemaker or retired, were associated with a higher likelihood of CE infection compared to non-agricultural workers. Similarly, it was observed that "Having relatives with CE" yielded an adjusted odds ratio of 4.18 (95% CI: 1.77; 9.88), suggesting that infection is acquired within a rural "domestic" setting, thereby indicating a "soil-transmitted" rather than a foodborne infection [23]. In another study employing a qualitative-quantitative approach to systematize information concerning knowledge, perceptions, and practices associated with exposure to *Echinococcus granulosus* in families with a history of CE in Huancayo, Peru, interviews were conducted with the closest family member to the case with CE (with the potential involvement of others). This inquiry revealed persisting knowledge, perception, and practice deficiencies among interviewees regarding CE, without reporting the prevalence of CE among the interviewed relatives [24]. Furthermore, in a series comprising 32 subjects diagnosed with CE in Catania, Italy, a robust correlation was observed between exposure to risk factors and the presence of more CE-affected individuals among patients’ relatives. This could potentially be attributed to the sharing of occupational or recreational risk factors between patients and their families [25]. In another cross-sectional study conducted in five provinces of western China involving 1500 pastoralist families, a prevalence of 1.6% for CE was ascertained among the surveyed families, with Figs reaching 2.5% in Tibet [26]. Finally, in other prevalence study undertaken in the northwest, north central, and northeast regions of Libya, in which 20,220 people were screened by portable ultrasound, 339 of them (1.7%), were diagnosed with CE [27]. Hence, it is of significance to generate information concerning familial clusters of CE, as there may exist families dwelling in environments where the parasite’s life cycle persists due to customary and cultural practices (such as domestic slaughtering of ungulates and the disposal of infected viscera into the environment), which remain unaltered despite awareness of the disease [28]. Nevertheless, the finding of practically all active lesions points to the opportunity to interrupt the transmission cycle for the disease independent of the type of cysts, which in this experience were mainly CE2 and CE3a.

In such contexts, there is a pressing need to intensify health education measures aimed at behavior modification and the promotion of canine deworming, among other interventions.

A striking finding was the low positivity of the Immunodiagnostic tests; however, although its yielded negative results in over 70% of cases, it is important to consider that 28.6% of the cases was CE1, and there is some evidence which support that CE1 cysts can be seronegative [29,30].

The novelty of this proposition lies in its regional scope, which is distinctive at a national level, encompassing a substantial number of cases. The prevalence results are noteworthy; according to our findings, within a familial cohort wherein a patient undergoes surgical intervention for CE, it is possible that one in six related family members could exhibit a similar condition, unbeknownst to them.

Among the study’s limitations, it should be noted that the non-participation of 10.6% of family members of patients surgically treated for hepatic CE constitutes a potential source of measurement bias. No risk factors were determined because sample size is still small to determine associated factors with adequate internal validity. On the other hand, we did not include other type of cases, such as patients with abdominal echinococcosis treated with benzimidazoles neither thoracic echinococcosis; because we do not use benzimidazoles (although there is evidence supporting the fact that resolution of CE in a non-surgical way with albendazole is effective in asymptomatic carriers with CE1 or CE3a cysts [31]; because all of patients are send to us just for surgical treatment (some of them had already failed their albendazole treatment). On the other hand, we do not have access to thoracic echinococcosis patients.

In summary, we can conclude that a heightened prevalence of CE exists among relatives of patients who have undergone surgical intervention for this disease in the province of Cautín, located in southern Chile. Consequently, it appears reasonable to postulate that individuals sharing the same household environment as patients treated for CE are at risk of contracting the disease, so any family with a member with diagnosis of CE (operated or not), should be assessed as rule. This finding underscores the significance of environmental control and the screening of relatives of patients treated for CE.
